# A Tragic Event Recalled

**Published:** 1889-11

**Authors:** 


A TRAGIC EVENT RECALLED.—Under this title the following paragraph appears in the daily press:

"Mrs. Elizabeth Wells, the widow of Dr. Horace Wells, the discoverer of anæsthesia, died at her home in Hartford, Conn., a few days ago. The death of Mrs. Wells recalls numerous incidents connected with the discovery made by her husband in 1844, when he was under thirty years of age. Of the three men who were with him when the gas was first administered two are still living, one of them being Col. Samuel Cooley, of Hartford, who was an intimate and trusted friend of Dr. Wells. Mr. G. Q. Colton, who made the gas and was present when it was taken by Dr. Wells, is living in New York city. The death of Dr. Wells which occurred in the Tombs in New York city, January 24, 1848, was a tragical event. He had been arrested on a charge of throwing vitriol on the garments of women, and the case seemed to be so clear against him that he committed suicide in preference to enduring the disgrace which the disclosures involved. The suicide was deliberately planned. In addition to administering chloroform to himself in quantities sufficient to kill him, he arranged a razor in such a way over an artery that when he was fully under the influence of the chloroform the instrument would descend, sever the artery and cause immediate death. The chloroform was administered by binding handkerchiefs, which had been saturated with it, over his mouth and head."

We publish the above in order that it may be contradicted if it should prove to be false, which we sincerely trust is the case.

